# Medical assessement of 3 years of activities in mahdia’s psychiatric department

**DOI:** 10.1192/j.eurpsy.2021.989

**Published:** 2021-08-13

**Authors:** M. Kacem, M. Khouaja, M.H. Aoun, L. Zarrouk

**Affiliations:** 1 Department Of Psychiatry, University hospital of mahdia, chebba, Tunisia; 2 Department Of Psychiatry, University hospital of mahdia, mahdia, Tunisia; 3 Psychiatry, University Hospital of Mahdia, Tunisia., Mahdia, Tunisia; 4 Department Of Psychiatry, University Hospital Of Mahdia, Tunisia., Psychiatry, Mahdia, Tunisia

**Keywords:** assessement, activities, psychiatric, department

## Abstract

**Introduction:**

The field of psychiatry extends from diagnosis to treatment, including prevention and various cognitive behavioral and emotional disorders.

**Objectives:**

To study the activity of Mahdia’s psychiatric department in order to improve its outcomes.

**Methods:**

This study was retrospective based on reporting data of the inpatients during 3 years (2016-2018) and then analyzing them.

**Results:**

This study involved 395 patients with an average age 36.6 years. The sex ratio M/F was 1.58. The prevalence of the disorders was more marked with the low socio-economic level, school failure and unemployment. 37% had a family psychiatry history and schizophrenia was the most common. 75.5% had a personal psychiatric history and 16.8% had a history of suicide attempt. Schizophrenia (28%), Bipolar Disorder (22.1%) and Depression (14.7%) were the main conditions. The majority 79.2% had irregular medical follow-up, 44% had poor therapeutic adherence. The majority 86.6% were hospitalized without consent. The most common reason was aggression and the average length of stay was 19.47 days. The mean duration of parenteral therapy was 4.38 days. Electro-convulsive therapy was indicated for only 16 patients. Typical antipsychotics were the most prescribed 37.4%. The exit treatment was monotherapy in 14.3% and polytherapy in 83.4%. The exit destination was home in 98% and the obligation follow-up was only indicated in 2.8% (11patients).

**Conclusions:**

This study is at the heart of psychiatric news with many questions around these coercive practices at legal and ethical level, particularly respect for freedom, legitimacy of these measures, patients’ safety and the quality of the treatments.

